# Personalized Surveillance Intervals for Intraductal Papillary Mucinous Neoplasm (IPMN): Multicenter Study Using Parametric Models

**DOI:** 10.1097/SLA.0000000000006702

**Published:** 2025-03-21

**Authors:** Claudio Ricci, Stefano Crippa, Johnathan Hee, Hyesol Jung, Gabriele Capurso, Marco Ferronato, José Lariño Noia, Myrte Gorris, Paula Ghaneh, Ihsan Ekin Demir, Nuzhat Ahmad, Max Heckler, Giulio Belfiori, Francesca Aleotti, Youngmin Han, Wooil Kwon, Gaetano Lauri, Matteo Tacelli, Olivier Busch, Kulbir Mann, Marina Migliori, Paolo Giorgio Arcidiacono, Helmut Friess, Charles M. Vollmer, Thilo Hackert, Marc Besselink, Riccardo Casadei, Jin-Young Jang, Brian Kim-Poh Goh, Massimo Falconi, Giovanni Marchegiani

**Affiliations:** * Department of Internal Medicine and Surgery (DIMEC); Alma Mater Studiorum, University of Bologna, Bologna, Italy; † Division of Pancreatic Surgery, IRCCS Azienda Ospedaliero-Universitaria Di Bologna, Italia; ‡ Division of Pancreatic Surgery, Pancreas Translational and Clinical Research Center, San Raffaele Scientific Institute, Milan, Italy; § Department of Hepatopancreatobiliary and Transplant Surgery, Singapore General Hospital and National Cancer Center Singapore, Singapore; ∥ Ministry of Health Holdings, Elementum, Singapore; ¶ Department of Surgery and Cancer Research Institute, Seoul National University Hospital, Seoul National University College of Medicine, Seoul, Korea; # Pancreatico/Biliary Endoscopy and Endosonography Division, Pancreas Translational & Clinical Research Center, San Raffaele Scientific Institute, Vita Salute San Raffaele University, Milan, Italy; ** IRCCS Azienda Ospedaliero-Universitaria di Bologna, Internal Medicine Unit, Italy; †† Endoscopy and Pancreatic Unit, University Hospital of Santiago de Compostela, Santiago de Compostela, Spain; ‡‡ Department of Surgery, Amsterdam UMC, University of Amsterdam, Amsterdam, The Netherlands; Cancer Center Amsterdam, Amsterdam, The Netherlands; §§ Royal Liverpool University Hospital, Liverpool, UK; ∥∥ Department of Gastroenterology, Klinikum rechts der Isar, School of Medicine, Technical University Munich, Munich, Germany; ¶¶ Division of Gastroenterology and Hepatology, University of Pennsylvania, Philadelphia, PA; ## Department of General, Visceral and Transplantation Surgery, Heidelberg University Hospital, Heidelberg, Germany; *** Department of Surgery, Division of General Surgery, Hospital of the University of Pennsylvania, Philadelphia, PA; ††† Department of General, Visceral and Thoracic Surgery, University Hospital Hamburg-Eppendorf, Hamburg, Germany; ‡‡‡ Surgery Academic Clinical Program, Duke-NUS Medical School, Singapore; §§§ Department of Surgical, Oncological and Gastroenterological Sciences (DiSCOG), Hepatopancreatobiliary and Liver Transplant Surgery, University of Padua, Padua, Italy

**Keywords:** branch-duct intraductal papillary mucinous neoplasm, parametric models, personalized surveillance

## Abstract

**Objective:**

The aim was to build a calculator for personalized surveillance of BD-IPMNs.

**Background:**

The interval time for surveillance of low-risk branch duct intraductal papillary mucinous neoplasms (BD-IPMNs) has not been established yet.

**Methods:**

The study included an international cohort of BD-IPMNs without worrisome features (WFs) or high-risk stigmata (HRS). IPMN evolution was defined as the occurrence of HRS or WFs. The derivation cohort comprised 60% of patients. The validation group comprised the remaining patients. A parametric survival model was developed in the derivation cohort using Akaike (AIC) and Bayesian (BIC) information criteria and c-index. A “k-fold” validation was used to measure the covariate effect on the accelerated failure time. Two models (“standard” and “conservative”) were built and validated using the second cohort.

**Results:**

The derivation and validation cohorts included 1992 and 1119 BD-IPMNs. The lognormal distribution best fitted the derivation cohort (AIC=2673; BIC=2718). The pooled *c*-index was 0.689 (0.668 to 0.718, 95% CI). The factors reducing the time needed for IPMN evolution were age [−2% (−1% to −3%) for each year] and cyst size [−2% (0% to −3%); for each mm]. The “conservative” model, called PANORAMA, was the only one that correctly classified the validation cohort (*c*-index 0.712 vs. 0.696; *P*=0.072).

**Conclusion and Relevance:**

The development of WF and HRS is influenced by the patient’s age and cyst size. After a prudential first control at 6 months, repeating a semestral/annual follow-up in this time frame could be too tight.

In recent years, several studies^1–4^ have demonstrated that branch-duct intraductal papillary mucinous neoplasms (BD-IPMN) without worrisome features (WFs) and high-risk stigmata (HRS) are at very low risk of progression toward malignancy. Nonetheless, the small subset of low-risk BD-IPMNs developing WFs or HRs during surveillance deserves special attention as it shapes the follow-up duration and timing.^5^ Available guidelines,^6–8^ in their latest iterations, still recommend multiple and repeated evaluations over time to capture radiologic changes requiring further diagnostic evaluation or therapeutic interventions, like endoscopic ultrasonography (EUS) or surgery. The overall length of surveillance and its intervals are based on the risk of the IPMN evolving over time. This evolution is measured with hazard ratio (HR) using Cox regression, which distills the risk into a constant baseline risk, shared across all patients, and relative risk, which describes how individual covariates influence the risk.^9^ However, the most obvious and rational approach should be to calculate the interval time of follow-up, estimating the time needed to observe relevant changes such as WFs or HRS occurrence.^10^ This approach reflects the knowledge that BD-IPMNs progress to malignancy through an established chain of events depicted by given clinical or radiologic features (WF and HRS) and can be explored using the “accelerated failure time” (AFT) model.^11^ The AFT model assumes that the impact of covariates is to speed up or slow down the disease progression. In contrast, traditional nonparametric or semiparametric models, such as the Kaplan–Meier or Cox Regression, assume that the disease progression is constant for the entire population and observation time.

AFT can be calculated only using a parametric survival method,^11^ which permits violating the assumption by exploring how the risk changes over time.

Through this approach, the present article aims to build a prediction model of BD-IPMN evolution over time. This model could serve as the backbone of a personalized surveillance strategy based on the evolution versus stability of the cysts.

## METHODS

### Patients and Eligibility Criteria

This study was approved by the local review board of each participating center and followed the Strengthening the Reporting of Observational Studies in Epidemiology (STROBE) Statement. Informed consent policy varied across centers, and in most cases, it was waived, given the use of historic de-identified data.^12^ Patients with presumed BD-IPMNs (radiologically or cytologically/histologically diagnosed) at low risk of degeneration, lacking WFs or HRS, were enrolled. The presumptive diagnosis was based on the presence of one or more dilated branch duct(s) communicating with a normal (5 mm or smaller) main pancreatic duct (MPD) on high-resolution axial imaging or EUS. The exclusion criteria were as follows: (i) a follow-up less than 12 months from diagnosis; (ii) a previous history of pancreatic resection; (iii) cysts highly suspicious for an alternative diagnosis such as mucinous or serous cystic neoplasms, pseudocysts, solid pseudopapillary tumors, and cystic neuroendocrine tumors; (iv) absence of relevant data for the study. The overall cohort of patients was divided into derivation and validation cohorts with a ratio of ~60/40. The derivation cohort was used to develop and internally validate the model. This cohort included all patients with presumed BD-IPMNs observed between January 2000 and December 2022 at nine tertiary centers in Western countries (Supplementary Table 1, Supplemental Digital Content 1, http://links.lww.com/SLA/F438). The external validation cohort comprised all patients with presumed BD-IPMNs observed between January 2006 and December 2017 at 2 tertiary centers in Eastern countries (Supplementary Table 2, Supplemental Digital Content 1, http://links.lww.com/SLA/F438).

### Data Collection, Definitions, Endpoints

Age, sex, and presence of high-risk individuals for familial pancreatic cancer (FPC)^13^ were registered. Clinical data included pancreas-related symptoms such as jaundice, acute pancreatitis, new onset, or worsening diabetes. Radiologic and endoscopic characteristics of the cyst were collected, including location and cyst size. The largest size was considered for the analysis when multiple cysts were present. Duration of surveillance was defined as the interval from diagnosis to the date of last follow-up, surgery, or death. Surveillance was performed according to each center’s policies in line with the International Association of Pancreatology (IAP) or European guidelines.^6–8,14^ The target event of the study was any relevant changes (new onset of WFs or HRS) occurring during follow-up. WFs and HRS were defined according to the latest update of the IAP guidelines.^6^ However, because of its retrospective nature, mural nodule size was not always available for analysis. For this reason, nonenhancing mural nodules were considered WF, and enhancing mural nodules were considered HRS.^14^ The final end point was to build a model to estimate the time needed to observe a relevant change.

### Statistical Analysis

The descriptive characteristics of patients were reported as number and percentage or median and interquartile (IQR) or range. The analysis was performed in 3 steps: (i) the model development using the derivation cohort; (ii) the internal validation splitting of the derivation cohort into 2 groups, “in-sample” and “out-of-sample”; (iii) the external validation using the model developed in derivation cohort to fit the survival data of training cohort. In the first step, the time-to-event model was built using the Western cohort, comparing Weibull, Exponential, Log-normal, and Log-logistic hazard distribution (Supplementary Figure 1, Supplemental Digital Content 1, http://links.lww.com/SLA/F438 and Supplementary Methods, Supplemental Digital Content 1, http://links.lww.com/SLA/F438). The effect of the covariates was reported using the accelerated failure time (AFT) in percentage.^15^ The covariates accelerate or decelerate the time to the event of interest, shortening or extending the time for clinical or radiologic relevant change of BD-IPMN. The best model distribution was selected, measuring the goodness of fit: the smallest value of Akaike (AIC) and Bayesian (BIC) information criteria indicates the best model.^16^ Moreover, the ability to discriminate patients with and without progression was measured with Harrel’s c-index.^17^ The discrimination power was considered sufficient, good, very good, strong, and perfect for the c-index around 0.6, 0.7, 0.8, 0.9, and 1, respectively.^17^ In the second step, the model was internally validated through a 5-fold cross-validation approach. Five times, the model was developed on nearly 80% of the derivation cohort and tested in the remaining 20% so that all patients were included in the test group once.^18^ Two different prediction models were obtained using the pooled values of coefficients after the 5-fold cross-validation. In the standard prediction model, the weight of the covariates was assumed to be equal to the mean effect observed in the derivation cohort. The 95% CI upper effect of covariate was used in the conservative model. Finally, the prediction model was externally validated in the Eastern cohort, measuring calibration^19^ and discrimination power.^20^ For this purpose, the prediction models were used to fit the survival data of the external cohort in a parametric survival function. All statistical analysis was performed using STATA (Statistical software for data science, release 18, Stata Corp, College Station, TX) and R-project (R version 4.2.2; R Foundation for Statistical Computing, Vienna, Austria). A *P* value<0.05 was considered statistically significant.

## RESULTS

### Overall Population

Table 1 shows the differences between derivation (n=1992) and validation (n=1119) cohorts. The median follow-up was 59 months (33 to 86). Considering the presence of several significant differences, the search for heterogeneity in demographics, clinical, and radiologic features between validation and derivation cohorts was satisfied.

**TABLE 1:** Characteristics and Outcomes of 3,111 Patients With BD-IPMN Without Worrisome Features Nor High-risk Stigmata Included in the Study

Characteristics of patients	Derivation cohort* n=1,992	Validation cohort† N=1,119	*P*
	n (%) or Median (IQR)		
Age, y	66 (58–73)	66 (59–73)	0.177
Sex			
Male	809 (40.6)	507 (45.3)	0.011
Female	1183 (59.3)	612 (54.7)	
FPC			
No	1869 (93.8)	1110 (99.2)	<0.001
Yes	123 (6.2)	9 (0.8)	
Size of the largest cyst, mm	12 (8–17)	11 (7–15)	<0.001
Site of lesion			
Head	507 (25.5)	398 (35.6)	<0.001
Body	390 (19.6)	425 (38)	
Tail	126 (6.3)	90 (8)	
Multiple	969 (48.6)	206 (18.4)	
Observation time, mo	55 (30-93)	62 (36-79)	0.007
Relevant change during surveillance‡			
No	1434 (72)	926 (82.7)	<0.001
Yes	558 (28)	193 (17.3)	
HRs during surveillance			
No	1974 (99.1)	1093 (97.7)	0.002
Yes	18 (0.9)	26 (2.3)	
WFs during surveillance			
No	1452 (72.9)	952 (85)	<0.001
Yes	540 (27.1)	167 (15)	
Surgical resection			
No	1896 (95.2)	1090 (97.7)	0.001
Yes	95 (4.8)	26 (2.3)	
PDAC occurrence			
No	1972 (99)	1114 (99.6)	0.141
Yes	20 (1)	5 (0.4)	

* Western centers.

† Eastern centers.

‡ Worrisome features or high-risk stigmata.

FPC indicates familiar pancreatic cancer; HR, high-risk stigmata; IQR, interquartile rage; WF, worrisome features.

### Model Selection Using the Derivation Cohort

Table 2 reports the parametric model performances, while Figure 1 and Supplementary Figure 2, Supplemental Digital Content 1, http://links.lww.com/SLA/F438, show the risk curve distribution of parametric models. The model that best fitted the derivation cohort data was the one with the lognormal distribution of the risk change. The lognormal model had the smallest AIC and BIC (2673 and 2718, respectively) and discrimination power of good quality (0.691). The σ value was 1.3 (1.2 to 1.4), suggesting that the BD-IPMN cohort had a high risk of progression during the first 5 years, but the risk decreased after without returning to zero. Sex, site of lesions, familial pancreatic cancer, and length of the follow-up did not influence the AFT. Factors significantly influencing the AFT were the patient’s age and the cyst size. With the increase in age, the time for change occurrence was reduced by 1.7% each year. With the increase in cyst size, the time for change occurrence was reduced by 2% for each year.

**TABLE 2:** Parametric Distributions of Failure Curve for BD-IPMNs in the Derivation Cohort

Parameters	Distributions*			
	Weibull (AFT)	Exponential (AFT)	Log-normal (AFT)	Log-logistic (AFT)
Age (for each year), %	1.7 (1.0–2.5)	1.9 (1–2.6)	1.7 (0.9–2.5)	1.7 (1–2.6)
Cyst size (for each mm), %	2 (0.7–3.1)	2.2 (0.9–3.4)	2 (1–3.5)	1.9 (0.8–3.2)
Ancillary parameter†	P= 1.1 (1.0– 1.2)	P= 1	σ=1.3 (1.2–1.4)	γ=0.7 (0.7–0.8)
Goodness of fit (AIC, BIC)	2705, 2750	2712, 2751	2673,2718	2688,2733
C statistic	0.691 (0.669– 0.714)	0.692 (0.669– 0.714)	0.691 (0.688– 0.713)	0.692 (0.670– 0.715)

The exponential transformation of the coefficient permits the obtaining of this value.

* sex, site of lesions, familial pancreatic cancer, and length of the follow-up did not influence the scale of failure function.

† parameter, which determines the shape of the risk curve.

AFT indicates accelerated failure time reported as a percentage; AIC, akaike information criterion; BIC, bayesian information criterion.

**FIGURE 1:**
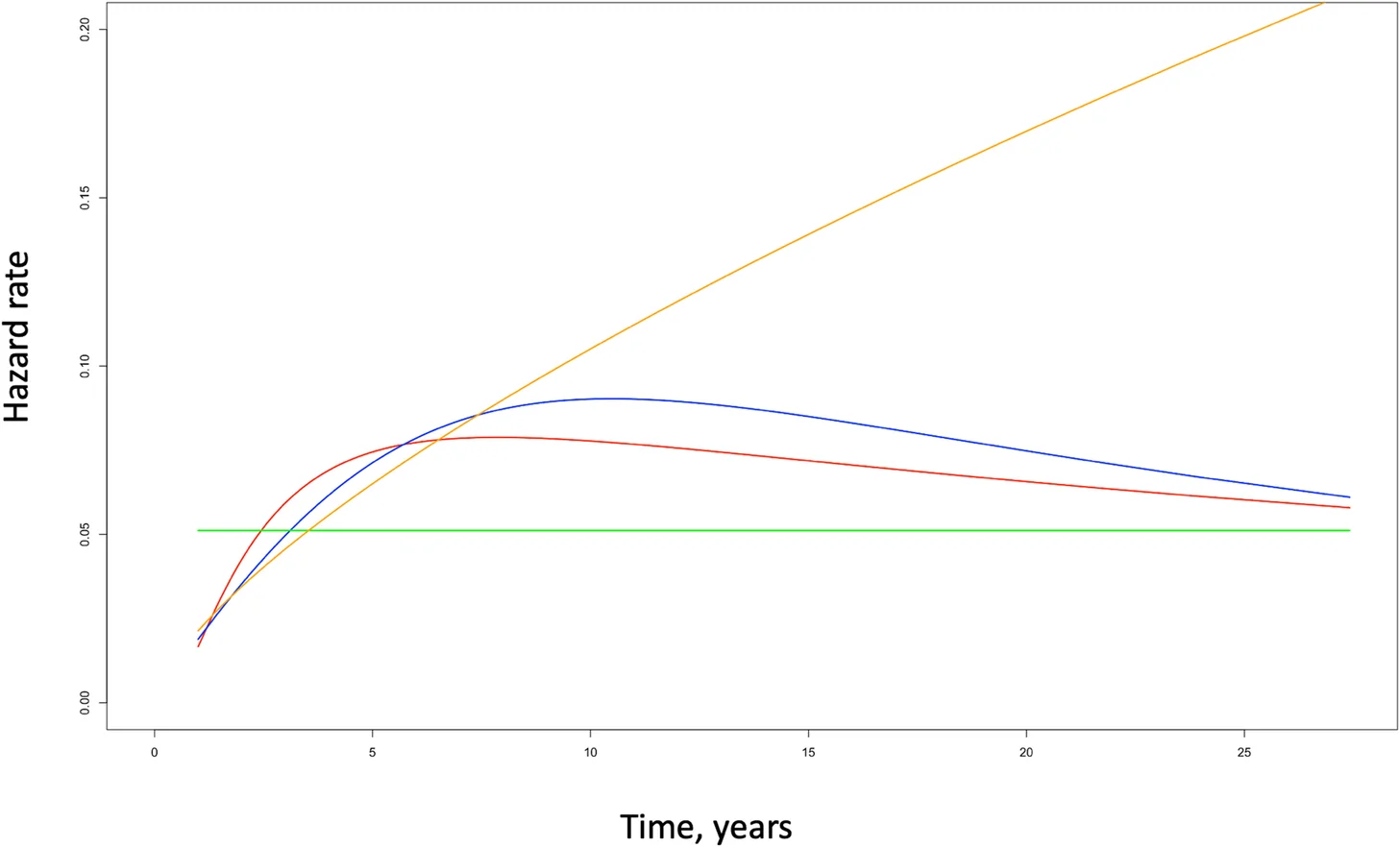
Hazard function. red = lognormal distribution; blue= logistic distribution; green = exponential distribution; orange= Weibull distribution.

### Model Development and Internal Validation Using the Derivation Cohort

In Table 3, the results of k-fold cross-validation are reported. In each of the 5 samples, the patient’s age at diagnosis and cyst size were always confirmed as the only factors influencing the AFT. Moreover, in each of the 5 samples, *C*-statistics were similar in both “in-sample” and “out-of-sample” cohorts, indicating a low risk of overfitting and good discrimination quality. The pooled data from the in-sample cohort suggested that the patient’s age and cyst size accelerated the time needed for change. As age increased, the time for change was reduced by 2% (1%–3%) for each year; as cyst size increased, the time for change was reduced by 2% (0%–3%) for each mm. The logarithmic transformation of these values and the use of the coefficient permitted the development of the 2 individual case-predicting algorithms. The standard model calculates the predicted median time for a change (in months) by solving the following equation: $\mathrm{time}=e^{\left(6.2+\left(\mathrm{size in mm}*-0.02\right)+\left(\mathrm{age in yrs}*-0.02\right)\right)}$ . In the conservative model, the effect of age and diameter was emphasized using the 95 CI superior coefficient for the 2 covariates: $\mathrm{time}=e^{\left(6.2+\left(\mathrm{size in mm}*-0.03\right)+\left(\mathrm{age in yrs}*-0.03\right)\right)}$ . Figure 2 summarizes conservative prediction models with a contour plot. Table 4 reports the predicted times for change divided into 3 categories based on time values in terciles: Q1, individuals with a high risk of change; Q2, subjects with intermediate risk; Q3, individuals with low risk. In the “Standard” model, patients at high risk of change did so after a median of 80 months (range 46–91), while those at intermediate and low risk after a median of 101 months (92–114) and 134 months (122–217), respectively. Using the “Prudential” model, patients at a high risk of change did so after a median of 33 months (range 14–40). Those at intermediate and low risk did so after a median of 46 (40–55) and 70 (55–200) months, respectively.

**TABLE 3:** Modelling for Accelerated Failure Time (AFT) in BD-IPMNs Using the Derivation Cohort

	k-fold cross-validation					
	1	2	3	4	5	Pooled (95% CI)
Training set, n	1547	1574	1582	1537	1590	
Age (for each year), %	2 (1–3)	1 (0–2)	2 (1–2)	2 (1–3)	2 (1–3)	2 (1–3)
Larger cyst diameter (for each mm), %	2 (1–4)	2 (1– 4)	2 (0–3)	2 (0–3)	2 (0.1–3)	2 (0–3)
Constant	6.3	5.9	6.3	6.2	6.1	6.2
C statistics (95% CI)	0.699 (0.674– 0.724)	0.674 (0.649– 0.701)	0.689 (0.664– 0.713)	0.691 (0.665– 0.717)	0.684 (0.659– 0.710)	0.689 (0.668– 0.718)
Test set, n	444	418	409	455	402	
*C* statistics (95% CI)	0.653 (0.602– 0.704)	0.741 (0.697– 0.786)	0.694 (0.644– 0.744)	0.694 (0.649– 0.740)	0.702 (0.654– 0.752)	0.702 (0.654– 0.752)

The time for change can be obtained by solving the following equation: time=EXP [6.2+ (size in mm *-0.02) + (age in y* -0.02)]; in the conservative scenario, the 95 CI superior CI can be used time=EXP [6.2+ (size in mm *-0.03) + (age in y* -0.03)] emphasizing the effect of age and cyst diameter.

**FIGURE 2:**
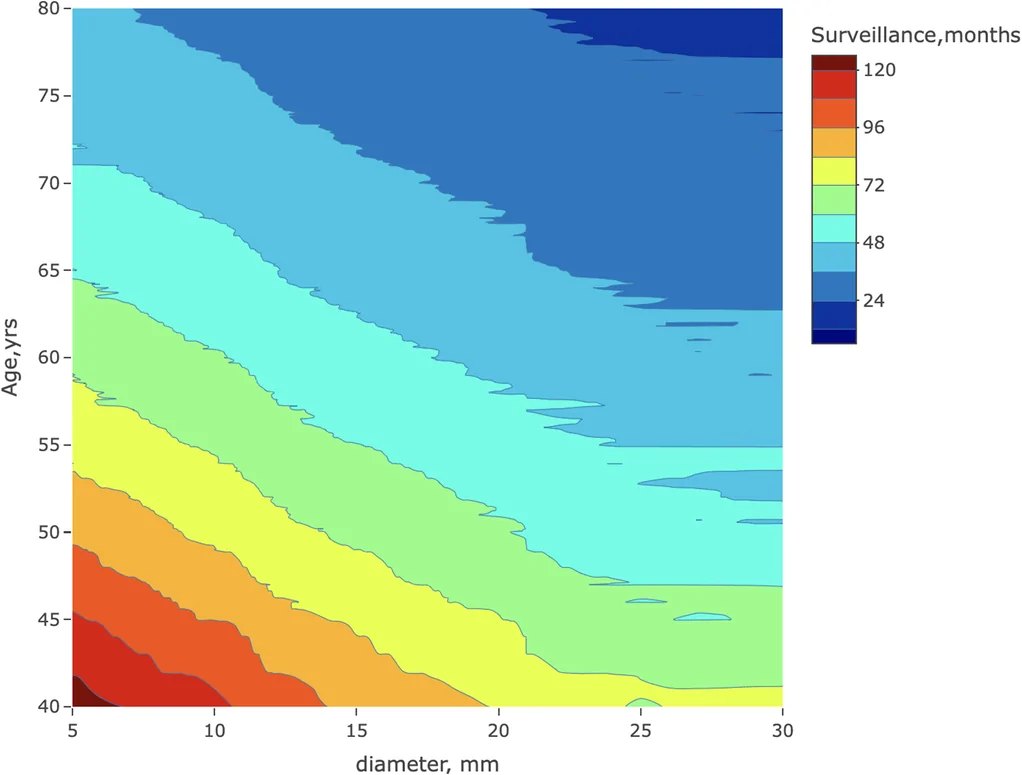
Contour plot of the conservative model. Time needed to observe a relevant change, according to variations in patients ‘age and cyst’s diameter. Individual time is accessible for any given patient at https://dpcg.nl/pancreascalculator/. The contour plots are derived from the following equations: $\mathrm{time}=e^{\left(6.2+\left(\mathrm{size in mm}*-0.03\right)+\left(\mathrm{age in yrs}*-0.03\right)\right)}$ .

**TABLE 4:** Predicted Interval Time Calculated in the Derivation Cohort Reported in Three Risk Categories

	Q1–high risk (n=702)	Q2–intermediate risk (n=626)	Q3–low risk (n=624)
PANORAMA “standard” model	80 (46–91) mo	101 (92–114) mo	134 (122–217) mo
PANORAMA “conservative” model	33 (14–40) mo	46 (40–55) mo	70 (55–200) mo

PANORAMA indicates Personalized Age and Size-based Surveillance for Low-Risk BD-IPMN of the Pancreas. The interval risk categories were obtained by solving the 2 equations and dividing the predicted interval time in terciles (Q1 <33%; Q2=33-66%; Q3>66%). Within each category, the time for change was reported with range.

### External Validation

Figure 3 shows the calibration curves, with the derivation and training models being similar and very close to the reference line, indicating that the observed and predicted risks are very similar. Table 5 shows the external validation process. First, a naïve model was developed using the training cohort population and regressing a parametric survival model based on lognormal distribution. In this model, the patient’s age and cyst size predicted the AFT similarly to that of the derivation cohort: (i) increasing age, for each year, the AFT was increased to 2% (1%–4%); (ii) increasing cyst size, for each mm, the AFT was increased of 5% (3%–7%). The external validity was confirmed by comparing the naïve model with the standard and conservative models. Using the standard model, the patients of the training cohort were classified into 3 different categories (namely high, intermediate, and low risk). However, the discrimination of these categories was not accurate as both intermediate and low-risk patients had AFTs that were not significantly different from those of high-risk patients. The suboptimal performance of the standard model was confirmed by a statistically significant difference with the naïve model 2 *C*-statistics (0.712 vs. 0.640; *P*<0.001).

**FIGURE 3:**
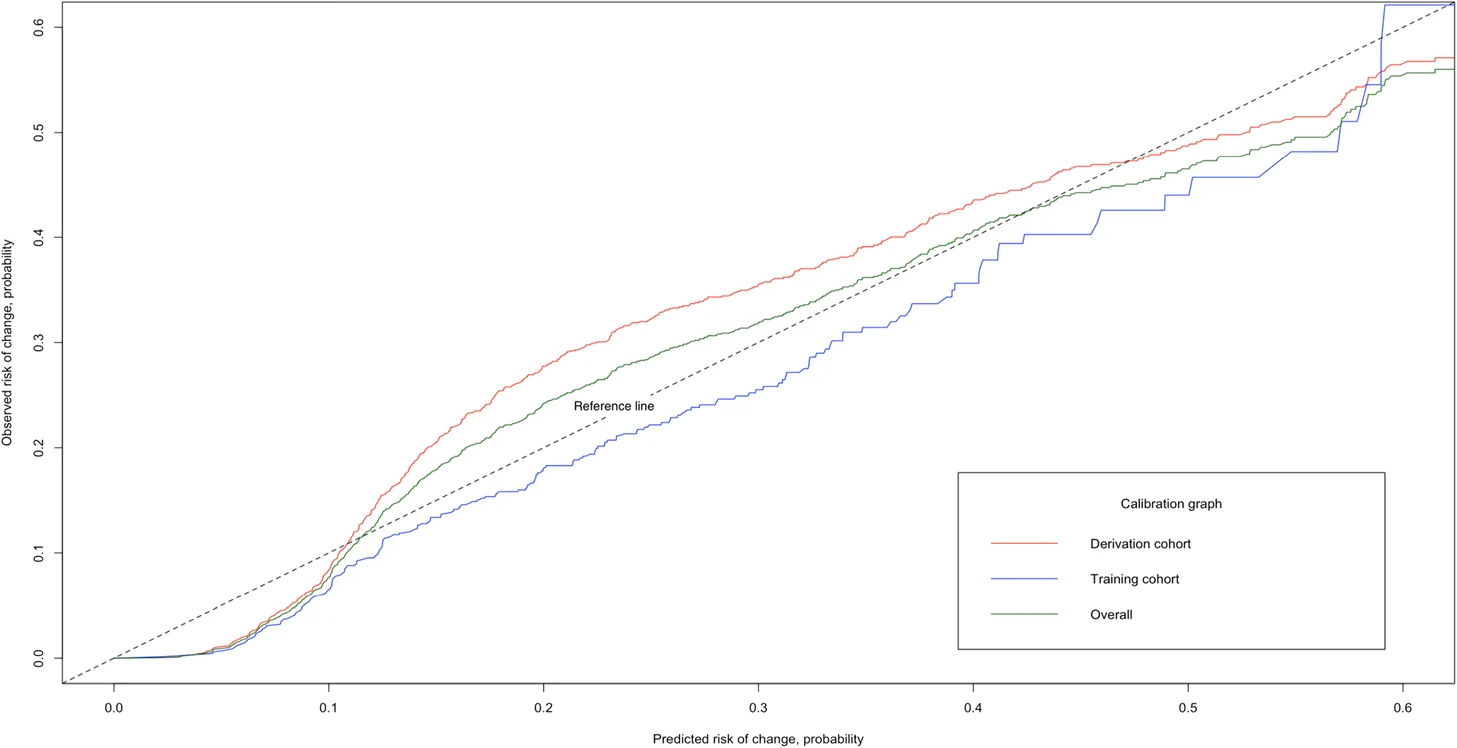
Calibration curve. The Y-axis represents the observed risk of relevant change; the X-axis represents the predicted pertinent change risk; the dotted line at 45 degrees indicates perfect calibration, as expected and observed probabilities are equal.

**TABLE 5:** Algorithms for Case-Predictions Interval Time Fitted in the Training Cohort

Parameters			
	Naive model (AFT)*	Standard PANORAMA model (AFT)†	Conservative PANORAMA model (AFT)†
High-risk category	—	Referent	Referent
Intermediate risk category, %	—	−16 (−213 to 56)	−60 (−12 to −129)
Low-risk category	—	−90 (−858 to 62)	−118 (−49 to −314)
Age, %	2 (1 to 4)	—	—
Diameter, %	5 (3 to 7)	—	—
Goodness of fit (AIC, BIC)	1152, 1177	1179, 1204	1162, 1187
*C* statistic; *P* ‡	0.712 (0.678 to 0.747); referent	0.640 (0.622 to 659); *P*<0.001	0.696 (0.664 to 0.728); *P*=0.072

* The naive prediction model was built using the age and diameter of the training cohort to calculate AFT.

† Standard and Prudential were developed using the equations developed in the derivation cohort, and these equations were used to classify the patients of the training cohort in high, intermediate, and low risk of progression.

‡ Harrel C statistic indicated the discrimination power, and the referent was the naïve model.

AFT indicates accelerated failure time reported in percentage; AIC, Akaike information criterion; BIC, Bayesian information criterion; PANORAMA, Personalized Age and Size-based Surveillance for Low-Risk BD-IPMN of the Pancreas.

On the contrary, using the conservative algorithm to classify the patients of the training cohort, the three categories well discriminated patients at different progression times. In detail, the time needed to change in patients classified as intermediate and low risk significantly decelerated by 60% and 118% compared with high risk. The conservative algorithm developed on the validation cohort has the same discrimination power as the model directly created using the original data of the training cohort (0.712 vs. 0.696; *P*=0.072) and, for this reason, can be considered externally validated. The individual case-predicting algorithms are available on the Excel spreadsheet in beta version (Supplementary material, Supplemental Digital Content 1, http://links.lww.com/SLA/F438) and at the following website: https://dpcg.nl/pancreascalculator/ after the publication of the manuscript.

## DISCUSSION

This international multicenter study provides the first online available model for personalized surveillance intervals in patients with low-risk BD-IPMN. This model determines the optimal surveillance interval based on 2 straightforward parameters, namely, the patient’s age and cyst size. For this reason, the calculator was called PANORAMA (“Personalized Age and Size-based Surveillance for Low-Risk BD-IPMN of the Pancreas”). It was developed and internally validated in a large cohort of tertiary referral Western centers and externally validated in a large cohort of Eastern centers with good discrimination power. The heterogeneity of the 2 populations regarding demographic, clinical, and radiologic factors confirmed the robustness of such a prediction model. However, the model was validated, excluding those BD-IPMNs undergoing rapid evolution. Therefore, it should be used in clinical practice only at centers of expertize for the management of pancreatic diseases and applied after the first 6 months of prudential observation, as recommended by the Kyoto guidelines.^6^ Of note, the PANORAMA use calculator is unsuitable for any BD-IPMN with WFs or HRS.

Due to the high prevalence of BD-IPMN in the general population (nearly 2%),^21^ surveillance in patients without WFs or HRS represents a contemporary “hot topic” with considerable implications for health care system organization and costs.^22^ In the last decade, several studies^1,5^ have demonstrated the indolent behavior of most of these cysts. Nonetheless, all available guidelines and related updates^6–8^ still recommend long-term follow-up in patients fit for surgery because the risk of progression is not null. These policies contrast the concept of the cost-effectiveness of oncologic screening, as they do not depict the actual risk of degeneration of IPMNs once considered in a dynamic fashion.

The current study uses a parametric survival approach to build a model that predicts the time needed to observe the relevant changes of low-risk BD-IPMNs, namely the development of WF and HRS. The first remarkable observation was that the change risk was not constant over time but decreased after 5 years, though never returning to zero and having a lognormal distribution of hazard function. This data is coherent with many observations associated with human cancers^23^ and clinical observations regarding BD-IPMN progression.^5^ Moreover, it confirmed that most low-risk BD-IPMNs did not significantly change within the first 5 years of observation, and therefore, repeating a 6-month/annual follow-up in this time frame could be too tight. In developing the prediction model in the derivation cohort, 2 main factors were associated with BD-IPMN evolution, namely cyst size and patient’s age. The evolution of cyst size over time is a well-known risk factor for malignancy in surgical series of IPMNs.^24^ Moreover, Han et al^24^ recently confirmed that a large cyst diameter at the baseline increased the risk of WF development during surveillance. In the present study, the time for WFs/HRS occurrence was shorter by 2% (1%–3%, months) for each mm in cyst size. This observation seems logical because BD-IPMNs can be assimilated to a hollow spheroid internally covered by an epithelium “at risk of change”; therefore, the larger the size, the larger the number of cells at risk.^25,26^ Moreover, the size at diagnosis estimates the time already passed from the initial occurrence, even if the cyst was undiagnosed. In other words, the larger the diameter, the older the cyst, thus increasing the risk of change. Also, the effect of age on WFs/HRS development seems to be aligned with epidemiological, clinical, and biological knowledge about BD-IPMNs. Prevalence and incidence studies^27–29^ showed that the median size of pancreatic cysts increases with age. Another prevalence study^21^ demonstrated that the standardized prevalence of WFs and HRS increases with age. In a molecular study, Omori et al^30^ reported that BD-IPMN and PDAC in the same patient shared several gene mutations, demonstrating a time-dependent progression from adenoma to carcinoma driven by somatic mutations. Finally, in a simulation model developed by Koopmann et al,^31^ the overall risk of PDAC in patients with BD-IPMNs increased with age. In the present effort, the PANORAMA calculator was built by solving the parametric survival equation after k-fold internal validation. In the conservative version, the effects of age and size were emphasized to 95% CI, assuming the worst scenario as the target of surveillance. In other words, the conservative PANORAMA model was based on the worst-case scenario to obtain an interval time that could capture the changes of evolving BD-IPMNs. Predictably, the conservative version of the calculator fits the data of the external validation cohort better than the standard one. The surveillance interval times obtained were sound and in line with the IPMN progression time reported by Koopmann et al.^29^ For instance, the conservative PANORAMA calculator suggests that a 75-year-old patient with a BD-IPMN of 30 mm in size could be followed every 2 years rather than every 6 to 12 months as recommended by the current guidelines.^6,8^ Moreover, if the same patient has a BD-IPMN of 15 mm, the PANORAMA calculator recommends an observation every 3 years rather than after 12 to 18 months.^6,8^ These recommendations should, however, be applied with caution and only by clinicians with high expertize in BD-IPMN management. Thus, it is always advisable to maintain a prudential first follow-up 6 months after the initial diagnosis in all presumed BD-IPMN to allow for an adequate test of time. Thereafter, in the continuing absence of WF and HRS, the application of the PANORAMA calculator might have a beneficial impact on health care costs without impacting patient safety.

The present study has several limitations. First, its retrospective nature could produce intrinsic bias. Second, although all involved centers are tertiary hubs for pancreatic cyst observation, detecting some BD-IPMN progressions could be suboptimal. Third, the study covered a long time for enrollment, during which the management of IPMNs evolved, resulting in a potential bias for the analysis. However, the management of low-risk BD-IPMN has only been slightly modified through different updates and versions of the IAP guidelines.^6–8^ Fourth, the risk of incorrect stratification of BD-IPMNs at baseline could generate a bias in interpreting their natural history. All patients with very early progression (6 mo) were excluded from the analysis to reduce this phenomenon, prudentially limiting the use of the calculator only after the first follow-up at 6 months, as recommended by the International guidelines.^6^ Another limitation is that the model suggested the interval time for BD-IPMN surveillance at the first diagnosis. However, it did not solve the dilemma of the follow-up modification when the changes were observed. In other words, the interval time should be modified when a WF occurs, and surgery is not performed. It should be remembered that the interval time suggested by PANORAMA was valid only for nonevolutive and low-risk cysts. Further studies applying the same AFT model in a population with WFs could clarify this key aspect. Fifth, the analysis did not include as a specific end point the occurrence of concomitant PDAC as such entity is not depictable without surgery. However, as previously described,^32^ the overall risk of cancer (both concomitant PDAC and malignant IPMN) was very low (<1%) once WF and HRS were lacking. The main strength of the study was that it is based on pragmatical cornerstones: (i) few low-risk BD-IPMNs will change, developing WFs or HRS and becoming at higher risk for cancer; (ii) an ideal approach to timely capture patients at higher risk of cancer development should be designed.

In conclusion, the conservative PANORAMA calculator accurately predicted the time needed for low-risk BD-IPMNs to evolve. This information allowed us to build a novel surveillance schedule for BD-IPMN without WF and HRS at diagnosis based on 2 pillars, namely age and cyst size. Further multicentric prospective studies are needed to confirm these data and reinforce the safety of this novel tailored surveillance protocol.
